# 

**Published:** 1881-06

**Authors:** 


					﻿TWE NTY-EIG HTH YEAR.
H-ÆTuI^’S
JOURNAL
HEALTH
E. H. GIBBS, A.M., M.D., Editor,
No. 141 EIGHTH STREET (Near Broadway), NEW YORK.
I TERMS: $2 a Year, or $1 for Six Months. Single nninher, 20 ctsJ
Entered aa Seoond etaea Matter In the Poet-Office in New York.
OF
				

